# The Status of Pro-social Tendency of Left-Behind Adolescents in China: How Family Function and Self-Esteem Affect Pro-social Tendencies

**DOI:** 10.3389/fpsyg.2019.01202

**Published:** 2019-05-28

**Authors:** Feifei Gao, Yuan Yao, Chengwen Yao, Yan Xiong, Honglin Ma, Hongbo Liu

**Affiliations:** ^1^Department of Health Statistics, School of Public Health, China Medical University, Shenyang, China; ^2^Department of Health Statistics, School of Public Health, Jinzhou Medical University, Jinzhou, China; ^3^Medical College, Qingdao University, Qingdao, China; ^4^Middle School of Ying-Li Town, Heze, China; ^5^Hospital of Xi-He Town, Chengdu, China

**Keywords:** left-behind adolescents, pro-social tendencies, self-esteem, family function, mediating effect, moderating effect

## Abstract

In China, adolescents are frequently left behind. To date, few studies have focused on the pro-social tendencies of left-behind adolescents and the relationship of family function, self-esteem, and pro-social tendency is yet to be examined. This study, therefore, aims to understand the status of pro-social tendency of left-behind adolescents and to explore the mediating and moderating roles of self-esteem in the relationship between family function and pro-social tendency. A large, school-based survey was conducted in three Chinese provinces. An analysis of covariance was first used to identify the differences in pro-social tendency between adolescents who were and were not left behind. We then analyzed the variance within left-behind adolescents using demographics, left-behind type, years of being left-behind, and caregiver related characteristics. A structural equation model was used to analyze the relationship of family function, self-esteem, and pro-social tendency, with bootstrapping used to explore the mediating role of self-esteem. Additionally, an ordinary least squares regression was used to examine the moderating effect of self-esteem. The results showed that the pro-social tendency of left-behind adolescents was lower than in non-left-behind adolescents (*F* = 15.11, *p* = 0.0001). Family function was positive related to pro-social tendency (*r* = 0.259), which had not only a direct effect on pro-social tendency (β = 0.254), but also an indirect effect through self-esteem (β = 0.071, bias-corrected 95% CI: 0.051:0.090; percentile 95% CI: 0.053:0.092). Additionally, 21.85% of the total effect of family function on pro-social tendency was mediated by self-esteem. Furthermore, self-esteem negatively moderated the relationship between family function and pro-social tendency (β = -0.208, *p* < 0.0001), such that the effect of family function on pro-social tendency became weaker as self-esteem increased. The current study verified the negative effect of being left behind on the social development of adolescents and contributed to the understanding of the importance of self-esteem in the relationship between family function and pro-social tendency. Interventions aimed at enhancing self-esteem should be developed and implemented in left-behind adolescents to promote wellness in the entirety of psychological and social outcomes.

## Introduction

In China, the rapid growth of the economy is contributing to an increasing amount of internal migrants moving from rural to urban areas to seek employment opportunities (Hu et al., 2008; Guang et al., 2017). Although some rural migrants have brought their families with to the cities (Nielsen et al., 2005), most parents opt not to take their children with them, due to financial constraints and the transient nature of the work in urban areas (Keung Wong et al., 2007; Gao et al., 2010). Those children are known as “left-behind children,” who stay in their rural home towns. It is estimated that over 61 million children in rural regions are currently growing up in the absence of their mother, their father, or both (Fellmeth et al., 2018). There is an urgent need for improved understanding of the impacts of family separation on the health and well-being of left-behind children. Adolescence is a critical stage of socialization in an individual’s life (Brand and Klimes-Dougan, 2010). Up to 20% of adolescents worldwide experience psychological or social disorders (World Health Organization., 2005). LBAs are separated from their parents over a long period of time and are cared for by their grandparents or other relatives, with some even taking care of themselves. Physical time with their parents is very limited and contact is generally maintained by telephone. While they may benefit from increased family income (Lu, 2015; Tao et al., 2016), the social and psychological cost as a result of sacrifices related to geographical and emotional closeness can’t be ignored (Chang et al., 2017; Fellmeth et al., 2018). Previous research has shown that LBAs are prone to feelings of being abandoned/unloved and felt confusion and worry, all of which may lead to negative consequences in socialization (Chang et al., 2017; Han et al., 2018).

Pro-social tendencies, defined as behaviors that are intended to benefit others, are regarded as social adaptations that play a crucial role in one’s social life (Jun, 2018). As a whole, this construct is one of the most important in regards to social functions and crucial aspect of adolescents’ positive development (Carlo and Randall, 2002; Liu et al., 2019). It is valued in most cultures, likely due to the relationship with harmonious human relationships (Calderón-Tena et al., 2011; Knight and Carlo, 2012). To date, few studies have focused on the influence of the left-behind experience on pro-social tendency among LBAs, with conflicting results from those that have. For example, some results suggested that LBAs were less likely to be pro-social (Chen et al., 2016) and that the negative effect on children tends to be higher for long-term parental migration than for short-term parental migration (Viet Nguyen, 2016). Contrarily, other research yielded no significant differences between left-behind children and those not left-behind (Xin and Xu, 2010; Ye, 2014). Additionally, the effect of parental migration varies across different areas and between different types of migration (Viet Nguyen, 2016). Therefore, it is imperative to elucidate the question of whether parent-child separation increases the risk of adverse effects on pro-social tendency and, if so, exploring the influential factors of pro-social tendency to address the psychological and social outcomes of LBAs.

Family function, defined as the degree of family cohesion and positive communication, measures the extent to which a family works as a unit and reflects a family member’s perception of and satisfaction with the functional state of the family (Shi et al., 2017). In a household with migrant parents, it is particularly difficult for a LBA to define family roles and boundaries (Zhou et al., 2018). As parents migrate to work with periods of separation from children, family function may be disturbed. Previous research has shown that LBAs tend to rate family function significantly lower than non-LBAs (Zhao et al., 2014), particularly those who are separated from their mothers alone or from both parents (Zhou et al., 2018). Families are the cornerstone of society and one of the main social environments for children. Results from previous research suggested that family function is positively associated with the pro-social tendency of the child (Mejia et al., 2006; Renzaho and Karantzas, 2010), pointing to the importance of the family environment in promoting pro-social behavior during the formative years of life. Those with good family function were more likely to have a high level of pro-social tendencies, suggesting that family function is a critical influencing factor in a child’s development and has an extensive impact on psychological and social outcomes. To date, however, little is known about the impact of family function on LBAs. Weakness of family function may seriously affect the socialization process of LBAs. Consequently, it has become a primary concern for researchers to find ways to decrease the risk of adverse effects of low family function on pro-social tendencies among LBAs.

Self-esteem is the evaluation the individual makes and maintains with regard to him or herself (Rosenberg, 1965). Self-esteem is an inner attitude at the center of the construction of personality and psychic balance that contributes to the development of adaptive processes over the course of one’s life (Doré, 2017). Individuals with low self-esteem may have fewer cognitive resources to cope with daily stressors, making them vulnerable to emotional exhaustion and social maladjustment. Previous studies have demonstrated that low self-esteem was more prevalent among LBAs than non-LABs (Tang et al., 2018) and that self-esteem is positively associated with pro-social tendencies (Yun and Lee, 2007), which indicates that those with high self-esteem are more likely to have pro-social tendencies (Fu et al., 2017; McChesney and Toseeb, 2018). Furthermore, self-esteem is considered to be a mediator between family function and internet addiction (Shi et al., 2017), as well as a mediator between social support/happiness and pro-social tendencies (Guo et al., 2014; McChesney and Toseeb, 2018) in other populations. Self-esteem also act as a moderator between family function and depression (Yoo and Chung, 2018) and between dispositional envy and pro-social tendencies (Yu et al., 2018). Both the relationship between family function and self-esteem and the relationship between self-esteem and pro-social tendency have been examined in other populations. To our knowledge, however, the relationship of family function, self-esteem, and pro-social tendency has yet to been studied in LBAs. Additionally, the mediating and moderating effects of self-esteem between family function and pro-social tendency have not yet been elucidated. While self-esteem is generally considered to be a relatively stable and enduring feature, it may change under certain circumstances (Zangirolami et al., 2018). In this sense, it is not a static phenomenon, rather it is a dynamic process that can be modified at any time. Previous research has demonstrated that interventions can improve self-esteem (Liu et al., 2015; Nosek et al., 2016; Zangirolami et al., 2018). Given the parent-child separation that inevitably arises under China’s existing social system, a quick improvement in the family function of LBAs may not be possible. Therefore, evidence demonstrating the mediating and moderating roles of self-esteem between family function and pro-social tendency indicates that pro-social tendency will likely be improved by intervening to increase self-esteem among LBAs.

Given the previous evidence and above mentioned concerns, the aim of this study is to understand the status of pro-social tendencies of LBAs and to analyze the relationship of family function, self-esteem, and pro-social tendency. Based on previous research, the following hypotheses have been put forward:

Hypothesis 1: The pro-social tendency of LBAs is lower than that of non-LBAs.

Hypothesis 2: Family function is positively related to the pro-social tendencies of LBAs.

Hypothesis 3: Self-esteem mediates the effect of family function on the pro-social tendencies among LBAs.

Hypothesis 4: Self-esteem moderates the direct relationship between family function and pro-social tendencies among LBAs.

## Materials and Methods

### Study Design and Sample

A cross-sectional study was conducted in April and May 2016, utilizing a population of adolescents in the Sichuan, Henan, and Shandong provinces of China. These highly populated provinces have been largely characterized by the exportation of labor forces to other major metropolitan regions in China. LBAs in these regions are quite prevalent and can be generalized to all of China.

A two-step random, stratified, cluster-based sampling technique was utilized for the current study. First, one city was randomly selected from each province (Nanchong in Sichuan, Zhoukou in Henan, Heze in Shandong). Next, one high school and two middle schools in rural areas were randomly selected within each sampled city. If the sampled school was small (number of students < 200), one additional school was randomly selected. In total, ten schools (three high schools and seven middle schools) were enrolled. All students in the selected schools were invited to enroll in this study. All study related materials were administered to the classes as a whole in their respective classrooms in a single, 30-min session without the presence of teachers.

A total of 9,675 students were recruited for this study, with 355 students either refusing to answer the survey or returning incomplete questionnaires, yielding a total *N* of 9,320 (96.33%) of whom we sought to categorize as LBAs or non-LBAs. Students were categorized as an LBA or non-LBAs by their response to the question, “Do one or both of your parents migrate to another place because of work for at least 6 months?” Students were relatively evenly split between the two groups, with 4,716 of the 9,320 students (50.60%) categorized as LBAs and 4,242 students categorized as non-LBAs.

### Measures

The pro-social tendency scale was taken from the social adjustment scale for adolescents (Zhou and Zhang, 2008). The scale consists of seven items measured on a 5-point Likert-type scale, rated according to the degree of agreement with which the respondent has experienced (1 = strongly disagree, 5 = strongly agree). Prior to performing any analyses, a CFA of the scale was conducted. The fit indices of the scale were acceptable: χ^2^ = 120.870, *p* < 0.0001, the GFI = 0.993, the AGFI = 0.975, the CFI = 0.967, and the RMSEA = 0.034. The composite reliability (0.8278) and the average variance extracted (0.4087) were also acceptable, indicating an acceptable internal quality of the model.

Family function was measured by the family APGAR, which is primarily used to assess one’s perception of family function by examining how he or she regards the relationships between family members (Smilkstein et al., 1982). It is comprised of five Likert-type items, each with three possible responses according to the frequency of feeling satisfied with each item, ranging from zero (hardly ever) to two (almost always). The final score is the sum of the scores from all five items, with higher final scores denoting better family function. The Chinese version of the scale has been widely applied in China, with excellent validity and reliability (Hai et al., 2017; Li et al., 2018). Results of a CFA demonstrated that the model of one factor was a good fit (χ^2^ = 133.027, *p* < 0.0001, GFI = 0.993, AGFI = 0.979, CFI = 0.931, RMSEA = 0.074), indicating the suitability of the scale in the current sample. The composite reliability (0.7484) and the average variance extracted (0.3744) suggested an acceptable internal quality of the model.

The self-esteem scale was selected from The Rosenberg Self-esteem Scale (Rosenberg, 1965). It is comprised of five items (e.g., “I think I have many advantages”), each with four possible responses according to the degree of agreement (1 = strongly disagree, 4 = strongly agree) and has been validated in adolescents to evaluate their self-worth and self-acceptance (Delgado et al., 2013; Romera et al., 2016). Overall self-esteem is demonstrated by a total score of all five items, with higher scores reflecting a higher level of self-esteem. The CFA fit indices of the scale in this study showed an acceptable fit (χ^2^ = 140.225, *p* < 0.0001, GFI = 0.975, AGFI = 0.926, CFI = 0.901, RMSEA = 0.076). The composite reliability (0.8058) and the average variance extracted (0.4559) were also acceptable, indicating good internal quality of the model.

### Statistical Analysis

Data analyses were conducted using SPSS 21.0 (SPSS China Corp., Shanghai, China) and Amos 20.0 software packages (SPSS Inc., Chicago, IL, United States). All statistical tests were two-sided and significance levels were set at α = 0.05. A CFA was performed to determine the internal structural validity of the instruments before exploratory analysis. The composite reliability and average variance extracted were calculated to evaluate the reliability and validity of the instruments. A composite reliability value at or above 0.60 suggests good reliability (Bacon et al., 1995). The average variance extracted value at or above 0.50 indicates adequate convergence, while between 0.36 and 0.50 may be acceptable (Fornell and Larcker, 1981). An ANCOVA was used to analyze the differences in pro-social tendency, family function, and self-esteem between LBAs and non-LBAs after adjusting for demographic characteristics and characteristics related to the caregiver. To identify the characteristics of pro-social tendencies among LBAs, an ANCOVA was also performed to explore the specific group variances of the pro-social tendencies after controlling for other demographics, left-behind characteristics, and caregiver characteristics.

Structural equation modeling was used to further verify the direct and indirect effects of family function on pro-social tendencies and self-esteem. Accounting for the categorical nature of the questionnaire variables and the descriptive results of the items, where the absence of normality was evident when some variables reached values well over zero in asymmetry and values of kurtosis were greater than 2 (Bollen and Long, 1994), ADF methods were used to calculate SEM with AMOS 20.0, which is widely recognized as an appropriate estimation procedure to handle non-normal multivariate data (Byrne, 2001; Huang and Bentler, 2015), particularly with a large sample size such as ours (*n* = 4716). The goodness-of-fit of the model was evaluated by the significance of χ^2^ (values above 0.05 indicate a good fit). As χ^2^ is highly susceptible to sample size, other indicators were also considered, namely the GFI, the AGFI, the CFI, and the RMSEA. A RMSEA value at or below 0.08 denotes a good fit, while values of 0.90 or above suggest a good fit for all other named indices (Tong and Bentler, 2013; Monroe and Cai, 2015). In addition, a bootstrapped estimate of 5000 samples was applied to evaluate the mediating roles of self-esteem between family function and pro-social tendency and both bias corrected 95% confidence intervals (CI) and percentile 95% CI were calculated (Biesanz et al., 2010).

In order to test the moderating effects of self-esteem on the relationship between family function and pro-social tendency, an OLS regression was used. The dependent variable (pro-social tendency) was regressed on the interaction term family function × self-esteem with the main effects of family function and self-esteem, as well as control variables (Dawson, 2014) entered into the equation. Since adding an interaction term can cause problems with multi-collinearity in a moderated multiple regression model, the interaction term was calculated from the *z*-standardized family function and self-esteem terms and then tested as a moderating effect of self-esteem. The variables were entered into regression model in four steps. In step 1, the demographic variables (age, gender, school type, study site, father’s education level, mother’s education level), left-behind characteristics (left-behind type, duration of being left-behind) and characteristics related with the caregiver (type of caregiver, age of caregiver, education level of caregiver, communication frequency between the caregiver and LBAs) were input as control variables. In step 2, the independent variable (family function) was added, and in step 3, the moderator (self-esteem) was added. The interaction term (*z*-family function × *z*-self-esteem) was entered into the model in step 4. The moderation effect is considered significant if the coefficient of the interaction term is significant. However, the size and precise nature of this moderation effect is not easy to derive from examination of the coefficients alone, therefore we plotted the effect in order to properly interpret it and used a simple slope test to calculate the predicted values of pro-social tendency under different conditions (a common method is to use values that are one standard deviation above and below the mean to show high and low values of family function and high and low values of self-esteem) and the predicted relationship between family function and pro-social tendency at different levels of self-esteem (Hu et al., 2018). Additionally, *f*^2^ was calculated to measure the size of the moderation effect, which is the ratio of variance explained by the interaction term alone to the unexplained variance in the final model (Aiken and West, 1991). A value of *f*^2^ at or below 0.02 indicates a small effect size (Dawson, 2014).

## Results

A total of 9,320 students were enrolled in the current study (30.87% from Sichuan, 47.96% from Henan, and 21.17% from Shandong). Approximately half, at 4,716 (50.60%), of the students were categorized as LBAs with the other 4,242 students categorized as non-LBAs. Most (65.65% of 4,716) of the LBAs were middle school students and 34.35% were high school students. LBAs were aged 10–18 years (Mean = 15.54; *SD* = 2.24), of which 51.05% were male and 48.95% were female. The majority of LBAs (70.06%) were left-behind by both parents, with 26.00% left by their father and 3.94% left by their mother. Additionally, 41.12% of LBAs were left behind for more than 10 years. Most who were left behind were under the supervision of their single parent (30.52%) or grandparents (62.67%), with 43.28% of caregiver 60 years or older and 34.87% between 40 and 60 years old. The education of LBA caregivers was generally very low, with 55.73% having completed primary school or less and 29.89% having completed middle school. Over half (56.82%) of the fathers of LBA had a middle school education and 54.18% of the mothers of LBA mothers had a primary school education or below. Most LBAs had regular communication with their caregivers, with 55.31% frequently communicating with their caregivers, 34.08% occasionally communicating with their caregivers, and 10.61% seldom or never communicating with their caregivers.

The mean value of pro-social tendencies among LBAs was 22.52 ± 4.53, compared to an average of 24.83 ± 4.36 for non-LBAs. To verify the hypothesis that LBAs may have lower pro-social tendencies than non-LBAs, we compared the pro-social tendencies between non-LBAs and LBAs using ANCOVA after adjusting for age, gender, study site, school type, and caregiver characteristics. The results showed that LBAs’ pro-social tendency was significantly lower than non-LBAs (*F* = 15.11, *p* = 0.0001). Furthermore, the family function and self-esteem of LBAs were also significantly lower than non-LBAs (see Table 1).

**Table 1:** Comparison of the main study variables between LBAs and non-LBAs.

Variables	Non-LBAs (*n* = 4,242)	LBAs (*n* = 4,716)	*F*	*p*
Family function	6.101 ± 2.418	5.511 ± 2.496	4.31	0.0379
Self-esteem	14.670 ± 2.582	14.375 ± 2.545	4.02	0.0451
Pro-social tendency	24.830 ± 4.366	22.522 ± 4.536	15.11	0.0001


### The Characteristic Analysis of Pro-social Tendencies Among LBAs

To identify the characteristics of pro-social tendencies among LBAs, an ANCOVA was used to explore the specific group variances of the pro-social tendency, after controlling for demographic, left-behind characteristics, and caregiver characteristics. The results showed that female LBAs scored significantly higher on pro-social tendency than males (*F* = 183.88, *p* < 0.0001). The mean pro-social tendency score of high school students was significantly higher than junior middle school ones (*F* = 5.20, *p* = 0.0226). Additionally, LBAs from the Henan province had more pro-social tendencies than those from the Sichuan provinces (*F* = 4.27, *p* = 0.0140). Additionally, the longer adolescents had been left behind, the lower their pro-social tendencies, LBAs who were left-behind for more than 10 years scored significantly lower on pro-social tendency those left behind for less than 10 years (*F* = 2.93, *p* = 0.0196). Furthermore, the higher the frequency of communication between the caregiver and LBAs, the higher the pro-social tendencies of LBAs (*F* = 38.32, *p* < 0.0001). No statistical differences were found in pro-social tendencies among other categorical items (*p* > 0.05, see Table 2).

**Table 2:** Characteristic analyses of pro-social tendency among LBAs.

Variables		*N*	$\bar{x}$ ± *s*	*F*	*p*
Gender				183.88	<0.0001
	Male	2,382	21.654 ± 4.779		
	Female	2,284	23.409 ± 4.103		
Age (years)				1.73	0.1579
	<12 years	107	23.047 ± 4.085		
	∼12 years	1,312	22.318 ± 4.839		
	∼14 years	1,560	22.359 ± 4.555		
	∼16 years	1,737	22.790 ± 4.291		
Study site				4.27	0.0140
	Sichuan	1,794	22.275 ± 4.899^a^		
	Henan	2,082	22.829 ± 4.098		
	Shandong	840	22.463 ± 4.823		
School type				5.20	0.0226
	Middle school	3,097	22.376 ± 4.647		
	High school	1,619	22.801 ± 4.305		
Left-behind type			0.94 ± 0.3910	
	Both parents	3,304	22.483 ± 4515		
	Father only	1,226	22.597 ± 4.594		
	Mother only	186	22.720 ± 4.549		
Care giver				0.66	0.5183
	Single parent	1,434	22.652 ± 4.634		
	Grandparents	2,945	22.459 ± 4.481		
	Others	320	22.675 ± 4.468		
Education level of father				0.51	0.6787
	Primary school or below	1,371	22.381 ± 4.512		
	Middle school	2,658	22.515 ± 4.484		
	High school	608	22.865 ± 4.689		
	College degree or above	41	22.439 ± 5.353		
Education level of mother				1.16	0.3240
	Primary school or below	2,522	22.347 ± 4.453		
	Middle school	1,738	22.697 ± 4.453		
	High school	353	22.918 ± 4.971		
	College degree or above	42	22.357 ± 4.953		
Education level of care giver				1.37	0.2491
	Primary school or below	2,597	22.408 ± 4.519		
	Middle school	1,393	22.544 ± 4.594		
	High school	537	23.106 ± 4.348		
	College degree or above	133	22.714 ± 4.638		
Age of care giver (years)				0.28	0.8397
	<20 years	223	22.502 ± 4.718		
	∼20 years	798	22.465 ± 4.768		
	∼40 years	1,630	22.615 ± 4.437		
	∼60 years	2,023	22.509 ± 4.500		
Communication frequency between caregiver and LBAs				38.32	<0.0001
	Frequently	2,571	22.923 ± 4.469^b^		
	Occasionally	1,584	22.220 ± 4.503^c^		
	Seldom or never	493	21.448 ± 4.683		
Duration of being left-behind				2.93	0.0196
	∼6 months	1,078	22.792 ± 4.402		
	∼1 year	436	22.365 ± 4.446		
	∼3 years	428	22.410 ± 4.648		
	∼5 years	833	22.360 ± 4.563		
	∼10 years	1,938	22.084 ± 4.835^d^		


*^*a*^The mean pro-social tendency of this group was significantly different from the second group; ^*b*^The mean pro-social tendency of this group was significantly different from the second group (*t* = 6.331, *p* < 0.0001) and the third group (*t* = 7.514, *p* < 0.0001); ^*c*^The mean pro-social tendency of this group was significantly different from the third group (*t* = 3.309, *p* = 0.0009); ^*d*^The mean pro-social tendency of this group was significantly different from the first group (*t* = 2.758, *p* = 0.0058), the third group (*t* = 2.269, *p* = 0.0233) and the fourth group (*t* = 2.256, *p* = 0.0241)*.

### Correlation Analyses of Family Function, Self-Esteem, and Pro-social Tendency

A Pearson correlation analyses revealed that the pro-social tendency of LBAs was positively correlated with family function (*r* = 0.259, *p* < 0.0001) and self-esteem *(r* = 0.206, *p* < 0.0001). In addition, family function and self-esteem were also positively correlated *(r* = 0.280, *p* < 0.0001).

### Testing for the Mediation Effect of Self-Esteem

**FIGURE 1:**
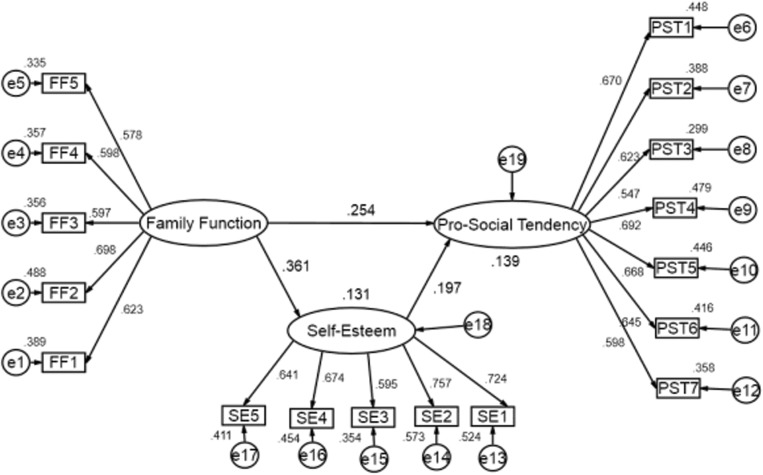
The structural equation model on the relationship of family function, self-esteem, and pro-social tendency among left-behind adolescents (family function, self-esteem, and pro-social tendency were used as latent variables; FF1–FF5, five items of family function; SE1–SE5, five items of self-esteem; PST1–PST7, seven items of pro-social tendency; e1–e17, the measurement error of each observed variable to estimate latent variable; e18–e19, the residuals that may affect the endogenous latent variables but not the exogenous latent variables; the path coefficient between two latent variable is the standardized regression weight, denoted as β; the path coefficient from the latent variables to each item is the standardized factor loading, which is denoted as λ; the coefficients marked next to the observed variables and dependent latent variables were squared multiple correlations).

Structural equation modeling was used to explore the direct and indirect effects of family function on pro-social tendency and self-esteem among LBAs. We used “family function,” “self-esteem,” and “pro-social tendency” as latent variables. Family function was measured by five items (FF1–FF5), self-esteem was also measured by five items (SE1–SE5), and pro-social tendency was measured by seven observed variables (PST1–PST7; see Figure 1). This model demonstrated that family function was positively related to self-esteem (β = 0.361, *p* < 0.001), explaining 13.1% variance of self-esteem (SMC of self-esteem = 0.131). Self-esteem had a positive direct effect on pro-social tendency (β = 0.197, *p* < 0.001), as did family function (β = 0.254, *p* < 0.001). Additionally, the results of a 5000 bootstrapped mediation analysis indicated that self-esteem significantly mediated the relationship of family function on pro-social tendency, with an indirect effect of 0.071 (bias-corrected 95%CI: 0.051:0.090; percentile 95%CI: 0.053:0.092). The explained variance of pro-social tendency was 13.9% in this model and the model fit was good (χ^2^ = 844.541, *p* < 0.0001, GFI = 0.967, AGFI = 0.956, CFI = 0.916, RMSEA = 0.036), determined by values of GFI, AGFI, and CFI greater than 0.90, and a RMSEA value less than 0.08. Based on the above analysis, the effect of family function on pro-social tendency was generated through two paths (See Table 3). First, the direct effect of family function on pro-social tendency was 0.254, accounting for 78.15% of the total effect (0.325). Second, there was an indirect effect of family function on pro-social tendency through self-esteem (0.071), which account for 21.85% of the total effect. Therefore, 21.85% of the total effect of family function on pro-social tendency was mediated by self-esteem. These results validate our hypothesis that self-esteem plays a mediating role in the relationship between family function and pro-social tendency.

**Table 3:** The effect size and proportion of paths in the total effect model.

Path	Effect size	Proportion (%)
Family function → pro-social tendency	0.254	78.15
Family function → self-esteem → pro-social tendency	0.071	21.85
Total	0.325	100.00


### Testing for the Moderating Effect of Self-Esteem

**FIGURE 2:**
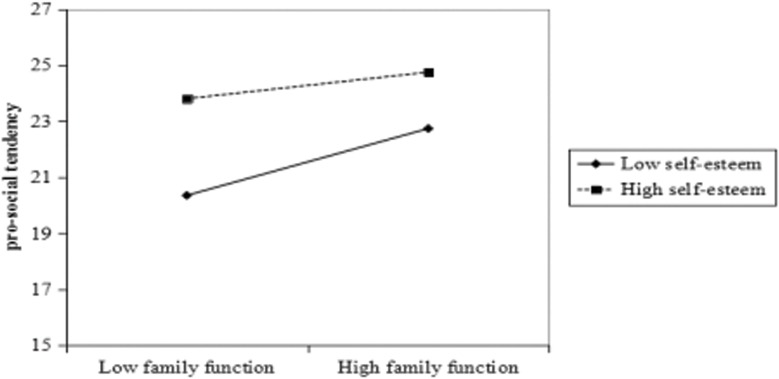
The moderating effect of self-esteem on the relationship between family function and pro-social tendency.

Table 4 presents the results of OLS regression analysis. The demographics, left-behind characteristics, and caregiver characteristics combined contributed to 6.11% of the variance of pro-social tendency, while family function and self-esteem accounted for 4.54 and 2.47%, respectively. Results from step 4 of the regression analysis suggest that self-esteem significantly negatively moderated the effect of family function on pro-social tendency (β = -0.208, *p* < 0.0001), accounting for an additional 5.22% variance of pro-social tendency. The value of *f*^2^ was 0.064, which is greater than 0.02, suggesting an acceptable size of the moderation effect. Therefore, these results supported the hypothesis that self-esteem negatively moderated the relationship between family function and pro-social tendency. The negative coefficient of the interaction term indicated that the effect of family function on pro-social tendency became weaker as self-esteem increased. Additionally, simple slope test results demonstrated that family function had a significant positive effect on pro-social tendency, both when self-esteem was low (β = 0.253, *t* = 15.43, and *p* < 0.0001) and high (β = 0.178, *t* = 8.42, and *p* < 0.0001). Figure 2 illustrates the interaction effect of self-esteem and family function on pro-social tendencies such that when family function moved from low to high, the high self-esteem individuals’ pro-social tendency increased significantly, however, the low self-esteem individuals’ pro-social tendency increased more steeply than those with high self-esteem. These results suggested that self-esteem could impact the effect of family function on pro-social tendency.

**Table 4:** The ordinary least squares (OLS) regression analysis for pro-social tendency.

Variables	Pro-social tendency			
	
	Step 1 (β)	Step 2 (β)	Step 3 (β)	Step 4 (β)
Age	0.099^b^	0.088^b^	0.078^b^	0.078^b^
Gender	0.195^b^	0.185^b^	0.206^b^	0.206^b^
School type	-0.035	-0.039	-0.044	-0.043
Study site 1 (Henan vs. Sichuan)	0.033^a^	0.003	0.009	0.009
Study site 2 (Shandong vs. Sichuan)	-0.006	-0.020	-0.026	-0.025
Left-behind type 1 (father vs. both parents)	0.001	0.004	0.004	0.003
Left-behind type 2 (mother vs. both parents)	0.019	0.025	0.024	0.023
Duration of being left-behind 1 (∼6 months vs. ∼10 years)	0.043^b^	0.046^b^	0.042^b^	0.042^b^
Duration of being left-behind 2 (∼1 year vs. ∼10 years)	0.036^a^	0.038^a^	0.036^a^	0.036^a^
Duration of being left-behind 3 (∼3 years vs. ∼10 years)	0.022	0.025	0.020	0.020
Duration of being left-behind 4 (∼5 years vs. ∼10 years)	0.035^a^	0.035^a^	0.031	0.030
Caregiver 1 (grandparents vs. single parent)	-0.020	-0.013	-0.013	-0.013
Caregiver 2 (others vs. single parent)	-0.004	-0.005	-0.003	-0.003
^c^Edu-caregiver 1 (middle school vs. primary school or below)	0.002	0.002	0.001	0.001
^c^Edu-caregiver 2 (high school vs. primary school or below)	0.028	0.020	0.020	0.019
^c^Edu-caregiver 3 (college degree or above vs. primary school or below)	0.008	0.007	0.008	0.008
Age of caregiver 1 (<20 years vs. ∼60 years)	-0.005	-0.015	-0.013	-0.014
Age of caregiver 2 (∼20 years vs. ∼60 years)	-0.025	-0.031	-0.028	-0.028
Age of caregiver 3 (∼40 years vs. ∼60 years)	-0.030	-0.027	-0.027	-0.028
^f^Communication frequency 1 (occasionally vs. frequently)	-0.103^b^	-0.053^b^	-0.048^b^	-0.050^b^
^f^Communication frequency 2 (seldom or never vs. frequently)	-0.119^b^	-0.049^b^	-0.041^a^	-0.040^b^
^d^Edu-father 1 (middle school vs. primary school or below)	0.008	0.001	0.001	0.001
^d^Edu-father 2 (high school vs. primary school or below)	0.021	0.011	0.007	0.006
^d^Edu-father 3 (college degree or above vs. primary school or below)	0.002	0.001	0.004	-0.004
^e^Edu-mother 1 (middle school vs. primary school or below)	0.033^a^	0.026	0.019	0.020
^e^Edu-mother 2 (high school vs. primary school or below)	0.018	0.012	0.009	0.010
^e^Edu-mother 3 (college degree or above vs. primary school or below)	-0.006	-0.009	-0.012	-0.011
Family function		0.230^b^	0.186^b^	0.187^b^
Self-esteem			0.168^b^	0.168^b^
Family function × self-esteem				-0.208^b^
*F*	11.68^b^	19.89^b^	24.10^b^	32.36^b^
Adjusted *R*^2^	0.0611	0.1065	0.1312	0.1834
Δ*R*^2^	0.0611	0.0454	0.0247	0.0522


*^*a*^*p* < 0.05; ^*b*^*p* < 0.01; ^*c*^Dummy variable of education level of caregiver; ^*d*^Dummy variable of education level of father; ^*e*^Dummy variable of education level of mother; ^*f*^Dummy variable of communication frequency between caregiver and LBAs.*

## Discussion

Despite the increased attention on the importance of family function to one’s physical and mental development, few studies to date have focused on the influence of parent-child separation on pro-social tendency among LBAs. Questions concerning the underlying mediating and moderating mechanisms of self-esteem between family function and pro-social tendency still remain largely unknown. Therefore, the current study aimed to understand the status of pro-social tendency and examined the relationships of family function, self-esteem, and pro-social tendency in a large number of LBAs in rural China. Furthermore, the current study explored the mediating and moderating roles of self-esteem on the relationship between family function and pro-social tendency. The main results of this study verified our hypotheses.

Consistent with previous research in China, the current study showed that approximately half of middle/high school students were left behind by one or both parents (Yuan and Wang, 2016; Fellmeth et al., 2018). The proportion of LBAs in China is higher than that in other countries, with 27% in Philippines and 36% in Ecuador (Yuan and Wang, 2016). The pro-social tendency of LBAs was lower than non-LBAs, with detriments particularly exacerbated in those who had been left behind longer, confirming the negative impact of parent-child separation and attachment interruption on the socialization process of LBAs in the critical period of development (Chen et al., 2016; Wang et al., 2017). Fortunately, our results suggest that frequent communication with caregivers may promote the development of pro-social tendencies among LBAs. Pro-social tendencies have been shown to be associated with a wide range of positive individual characteristics and outcomes, including empathy (Penner et al., 2005), agreeableness (Caprara et al., 2010), and peer acceptance (Layous et al., 2012). Furthermore, parental discipline techniques have been found to be important for building pro-social behavior during childhood (Flynn et al., 2015). Therefore, it is of particular importance to pay close attention to the pro-social tendencies of LBAs whose parents have been absent for a long time.

Male LBAs scored significantly lower on pro-social tendency than did females. Accumulating researches have demonstrated that males and females exhibit different methods of mental processing (Haferkamp et al., 2012; Gohier et al., 2013) and have found gender differences in social functioning (Huang et al., 2016). Seemingly, an unacceptable social environment could elicit opposite behaviors from females and males. Our results could, therefore, support the wider hypothesis of gender-based differences in mental processing. The mean pro-social tendency score of high school students was significantly higher than of middle school students and LBAs from the Henan province scored significantly higher on pro-social tendency than those from the Sichuan provinces. Even so, the practical value is not worth considering, as the difference of the mean score between groups was less than 1. These results are consistent with the findings of others (Eisenberg et al., 2015). Given our other results, we suggest that attention should be drawn to the pro-social tendency of LBAs, particularly males who have been left behind for many years.

Also consistent with previous research, results from the current study suggest that family function is positively associated with pro-social tendencies (Mejia et al., 2006; Renzaho and Karantzas, 2010). Those with good family function tended to have high levels of pro-social tendencies, suggesting that family function is an important influencing factor of pro-social tendency among LBAs. In accordance with previous research, the family function of LBAs suffered compared to non-LBAs, plausibly due to the geographical distance with parents and rare parent-child communication (Zhao et al., 2014; Zhou et al., 2018), suggesting potential social adaptation issues of LBAs as those who had lower family function tended to have lower pro-social tendencies. The current study also demonstrated that family function increased LBAs self-esteem, which in turn was positively related to LBAs’ pro-social tendency. That is, self-esteem mediated the relationship between family function and pro-social tendencies. Therefore, high level of self-esteem seems to be one of the explanatory mechanisms for why LBAs with good family function are less likely to have problem with pro-social tendencies. To the best of our knowledge, this is the first study to report such results in the literature. These findings are consistent with the social control theory, which posits that an individual’s attachment to others is a crucial element in the restriction of problem behaviors among adolescents (Hirschi, 1969). Indeed, LBAs with good family function leads to good family cohesion and positive communication with family members, as well as high family member intimacy. The relationships that LBAs build with key members in their life will increase their positive psychological state, which in turn will reduce the risk of psychological and social problems. These findings extend the prior research by illuminating why good family function can decrease problem in pro-social tendencies among LBAs. Some previous research on LBAs pro-social tendency has suggested the importance of paying attention to the influences of family function, whereas other research emphasized self-esteem. The current study was the first that integrated research from both areas to uncover LBAs pro-social tendency. This integrated model suggests that protective factors in the left-behind environment (e.g., family function) may enhance some protective intrapersonal traits (such as self-esteem), which in turn improve pro-social tendencies. In addition to the overall mediation results, each of the separate associations in the mediation model is noteworthy. For the first stage of the mediation process (family function→self-esteem), our findings support the notion that good family function is related to an increased in self-esteem in LBAs. This finding is consistent with the self-determination theory (Deci and Ryan, 2000) and the attachment theory (Ainsworth, 1989), both of which posit that good family member relationships play a vital role in one’s development. Indeed, LBAs with good family function are more likely to be people with good relationship with family members and psychological health, which in turn could prevent them from a series of psychological and social problems. For the second stage of the mediation model (self-esteem→pro-social tendency), self-esteem was positively associated with pro-social tendencies in LBAs. This finding is congruent with previous research (Zuffiano et al., 2016; Fu et al., 2017; McChesney and Toseeb, 2018), indicating that people with high self-esteem are more likely to have high pro-social tendency. This finding supports the notion that self-esteem is a motivating factor and important psychological resource for achieving positive social outcomes.

The other goal of this study was to examine whether the self-esteem could moderate the direct link between family function and pro-social tendency among LBAs. The results verified our hypotheses that self-esteem negatively moderates the relationship between family function and pro-social tendency. Family function had significant positive effect on pro-social tendency whether self-esteem was low or high, but it had a much greater impact in LBAs with low self-esteem than it did in those with high self-esteem. Self-esteem may, then, affect the effect strength of family function on pro-social tendency. Indeed, the effect of family function on pro-social tendency became weaker as self-esteem increased. Therefore, self-esteem can be regarded as a plausible indicator to distinguish whether people with low family function could be at greater risk for psychological or social problems. In light of above analysis, we can presume that self-esteem mediated and moderated the effect of family function on pro-social tendency among LBAs.

Consistent with previous research, LBAs had significantly less pro-social behaviors than non-LBAs (Chen et al., 2016; Viet Nguyen, 2016). Although the current study demonstrated that family function is positively related to pro-social tendencies, it is of little practical implication to improve LBAs’ pro-social tendency by improving family function, as this is a societal construct that would be difficult to improve in a short time under China’s existing social system. As long as there exists parent-child separation, it will inevitably affect the family function of LBAs. Both as a motivating factor and as an important psychological resource, self-esteem seems to mediate and moderate the effect of family function on pro-social tendency. Once self-esteem is improved, the adverse effect of low family function may be offset somewhat and pro-social tendencies will then be promoted, due to the positive relationship of self-esteem on pro-social tendency and the weakened impact of the adverse effect of low family function on pro-social tendency. Importantly, self-esteem is not a static phenomenon, but rather a dynamic process that can be developed and managed (Liu et al., 2015; Zangirolami et al., 2018). Interventions such as physical activity and mindfulness-based programs, which have been designed to enhance self-esteem, have been effective with other groups with a set of psychological and social outcomes positively changed (Nosek et al., 2016; Jalali et al., 2018; Zangirolami et al., 2018). It is plausible that if these interventions are also effective with LBAs, it will be of great practical significance to improve their pro-social tendencies. Therefore, it is our contention that strategies of enhancing LBA’s self-esteem should be developed and implemented in China as soon as possible, particularly in males who have been left behind for several years.

Based on the above analysis, our findings have important implications to the management of LBAs. First, the current study demonstrates that parent-child separation has negative effects on family function and pro-social tendencies among LBAs that and self-esteem may contribute to the ability to use psychological resources to help combat low family function and promote pro-social tendencies. These results promote the importance of self-esteem in dealing with the consequences of poor family function, allowing individuals to maintain a positive and healthy status. Furthermore, our findings offer a new perspective for LBA’s managers (such as teachers, or the government.) to combat the adverse effect of poor family function on pro-social tendency by providing LBAs with self-esteem development training programs (for example: a mindfulness-based program), particularly males that have been left behind for several years. Despite the many contributions made by the current study, there are limitations that could be improved in future research. For example, study utilized a cross-sectional research design that cannot determine causality and data was collected during a fixed period. To combat these limitations, future studies should consider utilizing a longitudinal design to track the pro-social tendency of LBAs through their growth trajectory. Additionally, we relied on self-report, which can be subject to information bias. Future studies should collect additional information from the at-home parents, caregivers, teachers, and peers. Furthermore, the current study only recruited rural students. To be able to generalize results, students in non-rural areas should also be included in future studies. Additionally, the current study only researched family function as it influences pro-social tendencies and only measured certain demographic, left-behind characteristics, and caregiver characteristics as potential confounding variables. Additional risk factors and confounding variables, such as socioeconomic factors, should be included in future studies. In spite of the above limitations, the present study provides a preliminary and novel understanding of the underlying mechanisms of the relationships between family function, self-esteem, and pro-social tendencies among LBAs.

## Conclusion

In conclusion, the present study adds to the existing literature suggesting that parent-child separation has a negative effect on family function and pro-social tendencies among LBAs. Family function had a positive impact on self-esteem and pro-social tendency, while self-esteem had a significant positive effect on pro-social tendency. Furthermore, family function was found to have a positive direct and indirect effect on pro-social tendency, through self-esteem. Indeed, 21.85% of the effect of family function on pro-social tendency was mediated by self-esteem. Additionally, self-esteem negatively moderated the relationship between family function and pro-social tendency; the effect of family function on pro-social tendencies weakened as self-esteem increased. Seemingly, then, self-esteem appears to affect the impact of family function on pro-social tendencies. The current study provides an initial insight into the potent mechanisms of the relationships between family function, self-esteem, and pro-social tendency among LBAs, which has, to the best of our knowledge, not yet been examined. These results provide insights into the importance of self-esteem in LBAs and provide a theoretical and practical basis to the managers of LBAs to develop targeted interventions to improve LBA’s self-esteem, to aid in the successful coping of poor family function so that they may maintain a positive and healthy social status. Because of the cross-sectional design of the present study, we intend to design a longitudinal study to confirm the dynamic relationship among family function, self-esteem, and pro-social tendencies.

## Ethics Statement

This study was carried out in accordance with the Declaration of Helsinki. The protocol was reviewed and approved by the ethical committee group of Jinzhou Medical University. The study procedures were carried out in accordance with the ethical standards. No bio-markers or tissue were collected. Prior to the administration of any study related questionnaires, approval from the officials of sampled schools was obtained in writing and the written informed consent was obtained from the parents or legal guardians on behalf of the adolescents to participate in the study. Participation was entirely voluntary, confidential, and anonymous. Participants were informed that they were free to withdraw from the study at any time.

## Author Contributions

FG was involved in the study design, analysis, and interpretation of the data as well as the drafting and revising of the manuscript. YY participated in data acquisition, study design, and interpretation of the results. CY, YX, and HM provided help with the data collection, analysis, and interpretation. HL made substantive intellectual contributions to the interpretation of data and the draft of the manuscript. All authors have read and approved the final manuscript.

## Conflict of Interest Statement

The authors declare that the research was conducted in the absence of any commercial or financial relationships that could be construed as a potential conflict of interest.

## Abbreviations

ADF: asymptotically distribution-free
AGFI: adjusted goodness of fit index
ANCOVA: analysis of covariance
CFA: confirmatory factor analysis
CFI: comparative fit index
GFI: goodness of fit index
LBAs: left-behind adolescents
OLS: ordinary least squares
SEM: structural equation model
SMC: squared multiple correlation
RMSEA: root mean square error of approximation
